# Supplementary material for: Comparing kinetic versus stoichiometric priorities in hybrid models of CHO metabolism
Source: NPJ Syst Biol Appl. 2026 May 29;12:133. doi: 10.1038/s41540-026-00703-5 (PMC13550590; doi:10.1038/s41540-026-00703-5)
Supplement: Supplementary file 1 [file 41540_2026_703_MOESM1_ESM.pdf]

SUPPLEMENTARY INFORMATION

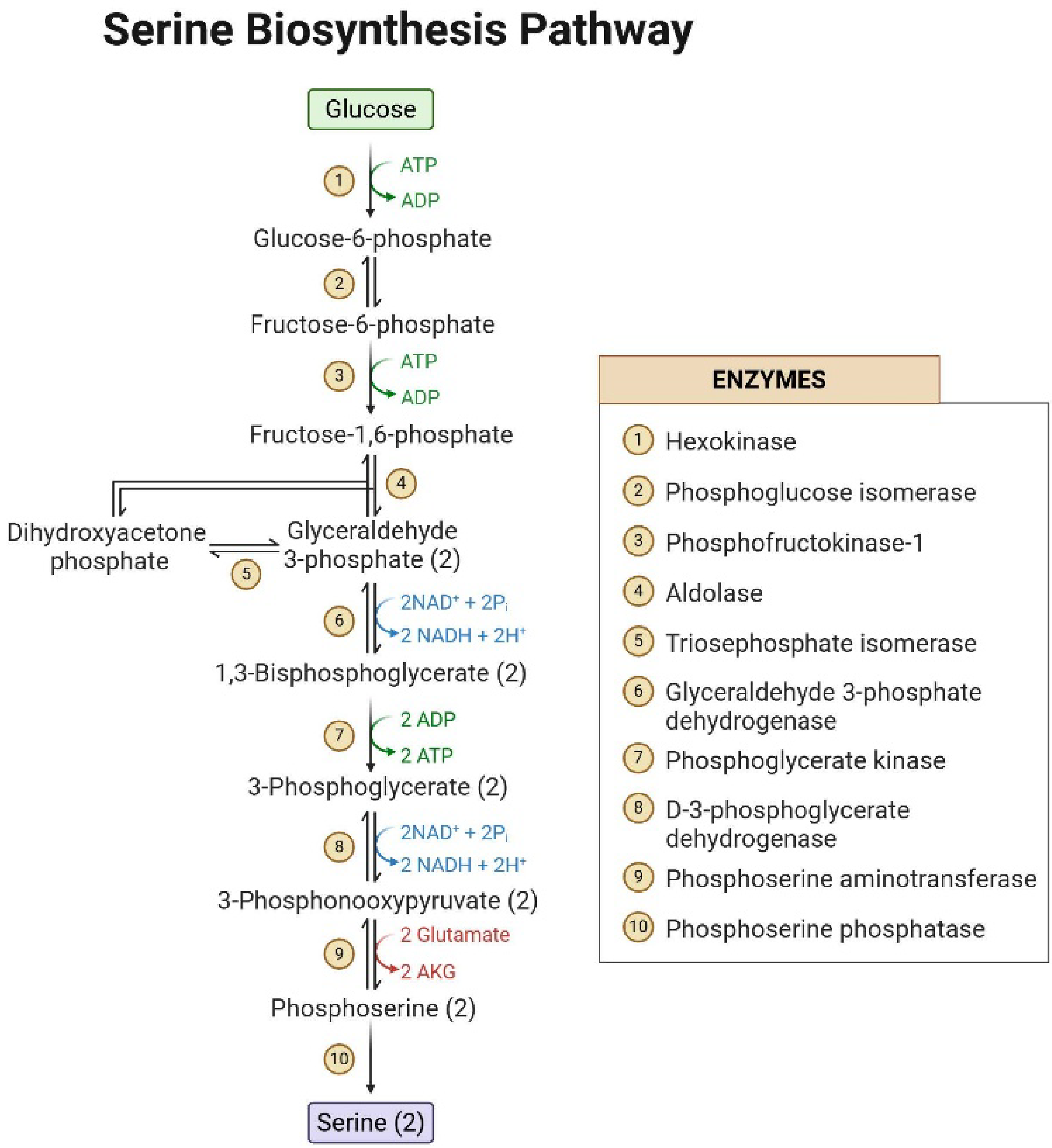

Supplementary Figure 1: Serine Biosynthesis Pathway. Individual reactions in the serine biosynthesis pathway, from which a condensed reaction expression was derived.
Created in BioRender. Betenbaugh, M. (2026) https://BioRender.com/e779cyh

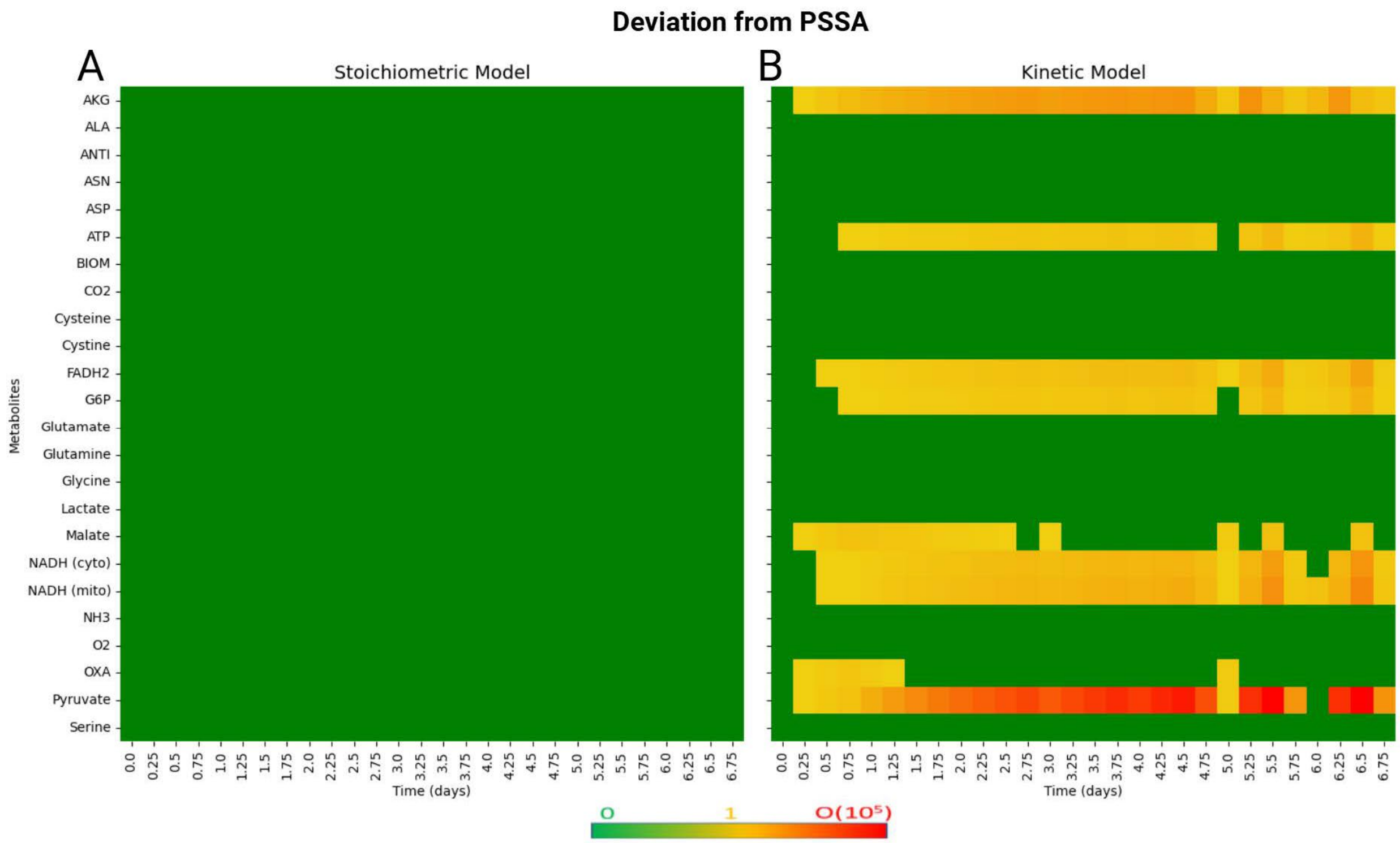

Supplementary Figure 2: Comparison of PSSA performance in stoichiometric-oriented vs. kinetic-oriented models. At each simulated time point, the S·v matrix is solved to evaluate the extent to which intracellular fluxes satisfy the pseudo steady state assumption (PSSA). **(A)** In the stoichiometric-oriented model, the fluxes are computed such that the PSSA is strictly enforced at each time point. The resulting heatmap shows that the net flux across each metabolite in the control dataset remains near zero, indicating full adherence to steady state conditions. **(B)** In the kinetic-oriented model, the fluxes from the kinetic expressions are fixed inputs, and therefore the PSSA cannot be fully satisfied. As a result, several metabolites show moderate to significant deviations from PSSA. Specifically, AKG, ATP, FADH2, G6P, malate, and oxaloacetate exhibit moderate deviations, while NADH and pyruvate, both in the cytosol and mitochondria, show substantial deviations.

Created in BioRender. Betenbaugh, M. (2026) https://BioRender.com/83dq76o

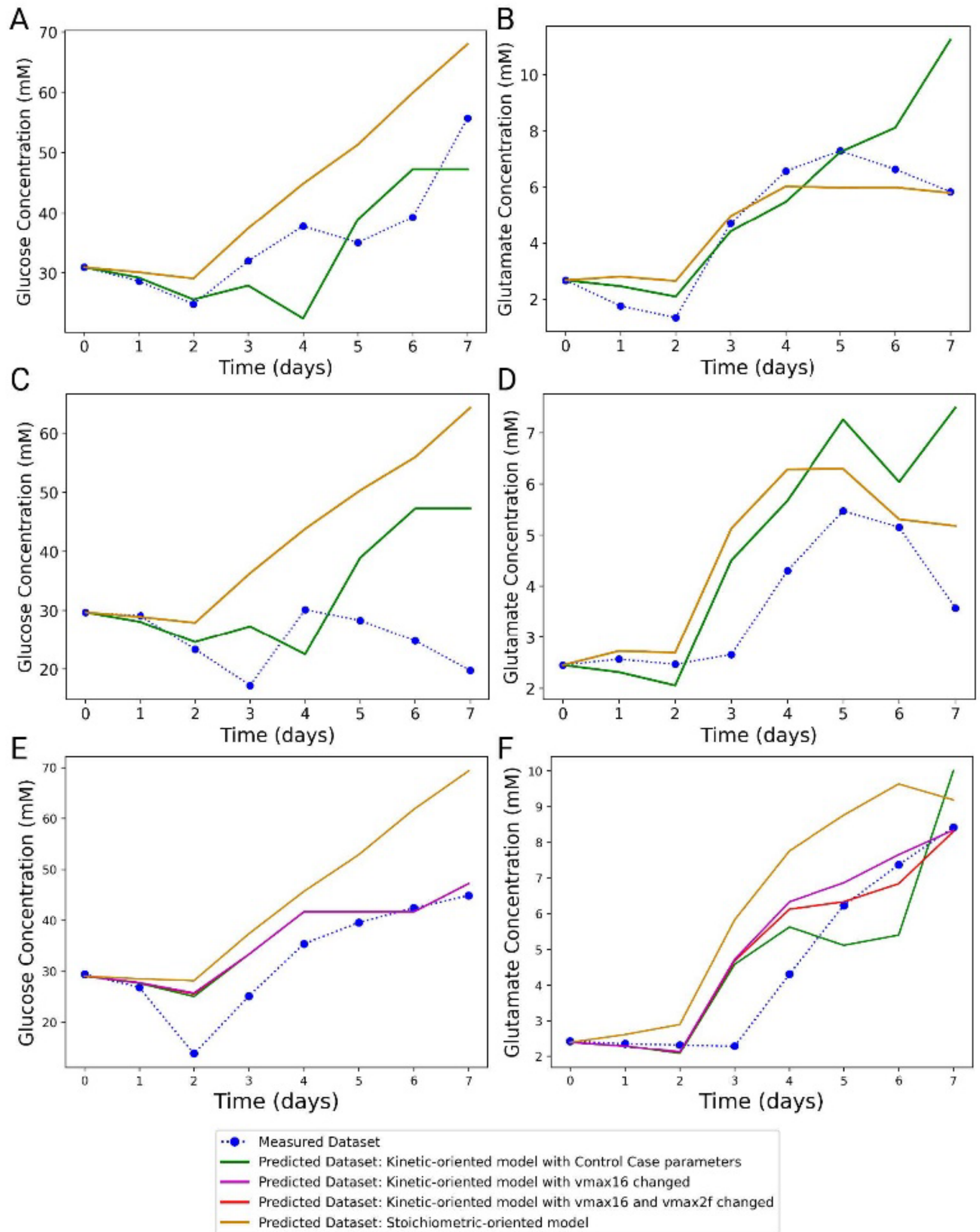

Supplementary Figure 3. KOM vs SOM predictive profile comparison for glucose (left side graphs) and glutamate (right side graphs) profiles for (A-B) Control case (Parameters were fit to this dataset) **(C-D)** Modified Initial Conditions- Alternate Dataset 1 (control case parameters used) **(E-F)** Lactate-Supplemented Case- Alternate Dataset 2 (control case parameters used, with modifications to *vmax16* and *vmax2f* parameters).
Created in BioRender. Betenbaugh, M. (2026) https://BioRender.com/6aa32v6

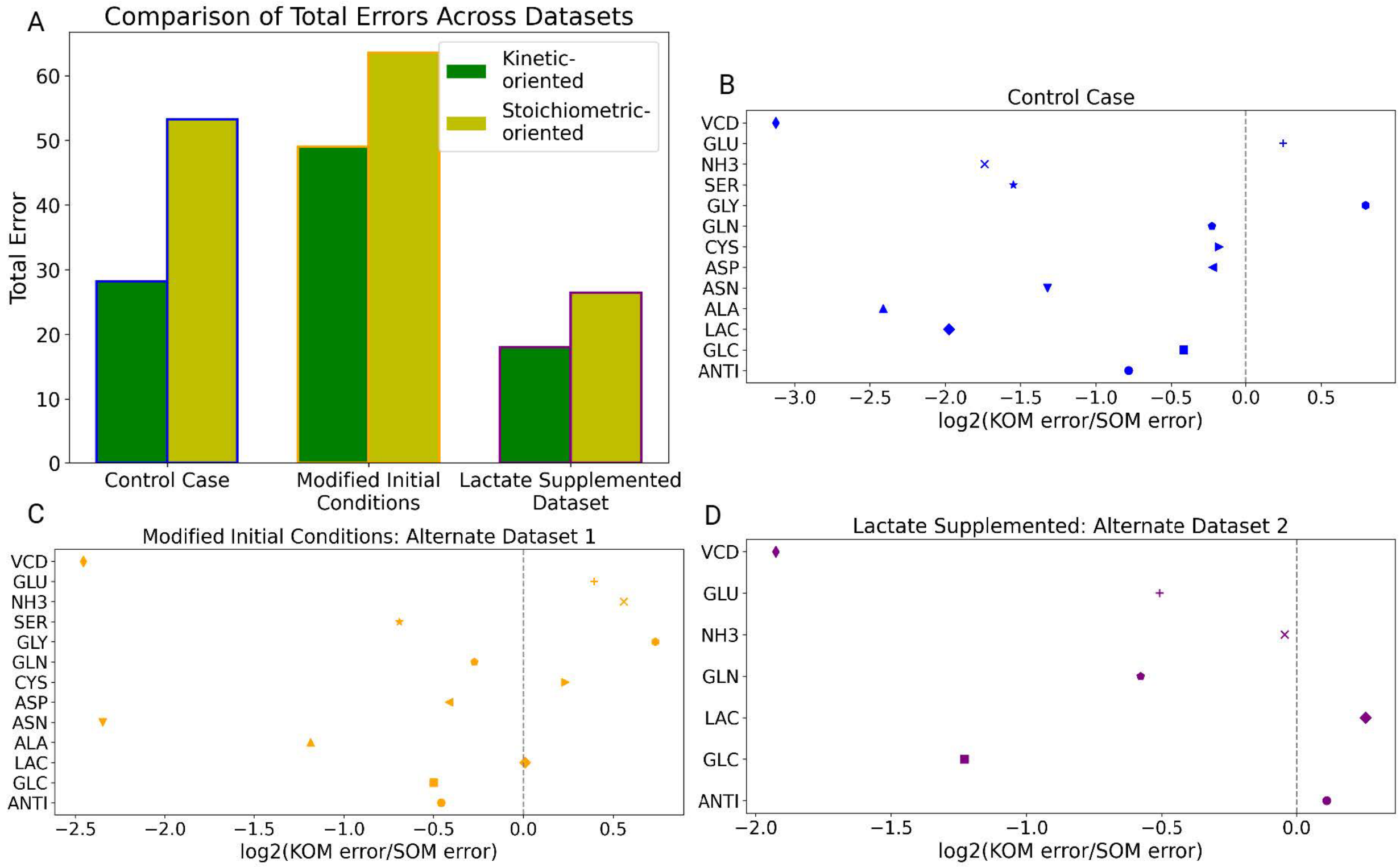

Supplementary Figure 4: Analysis of error between the kinetic-oriented model (KOM) and the stoichiometric-oriented model (SOM). (A) Total error for the KOM versus the SOM across all datasets, computed as the sum of errors for all metabolites and timepoints. **(B)** Error comparison for the Control Case, shown as log2 fold change of KOM error relative to SOM error for each metabolite. Most metabolites fall below zero, indicating lower error for the KOM. **(C)** Error comparison for the Modified Initial Conditions (Alternate Dataset 1), shown as log2 fold change of KOM error relative to SOM error. The majority of metabolites fall below zero, again favoring the KOM. **(D)** Error comparison for the Lactate-Supplemented Case (Alternate Dataset 2), shown as log2 fold change of KOM error relative to SOM error, shown only for those metabolites with measured data available. Most metabolites fall below zero, indicating reduced error with the KOM.

Created in BioRender. Betenbaugh, M. (2026) https://BioRender.com/cgw6byw

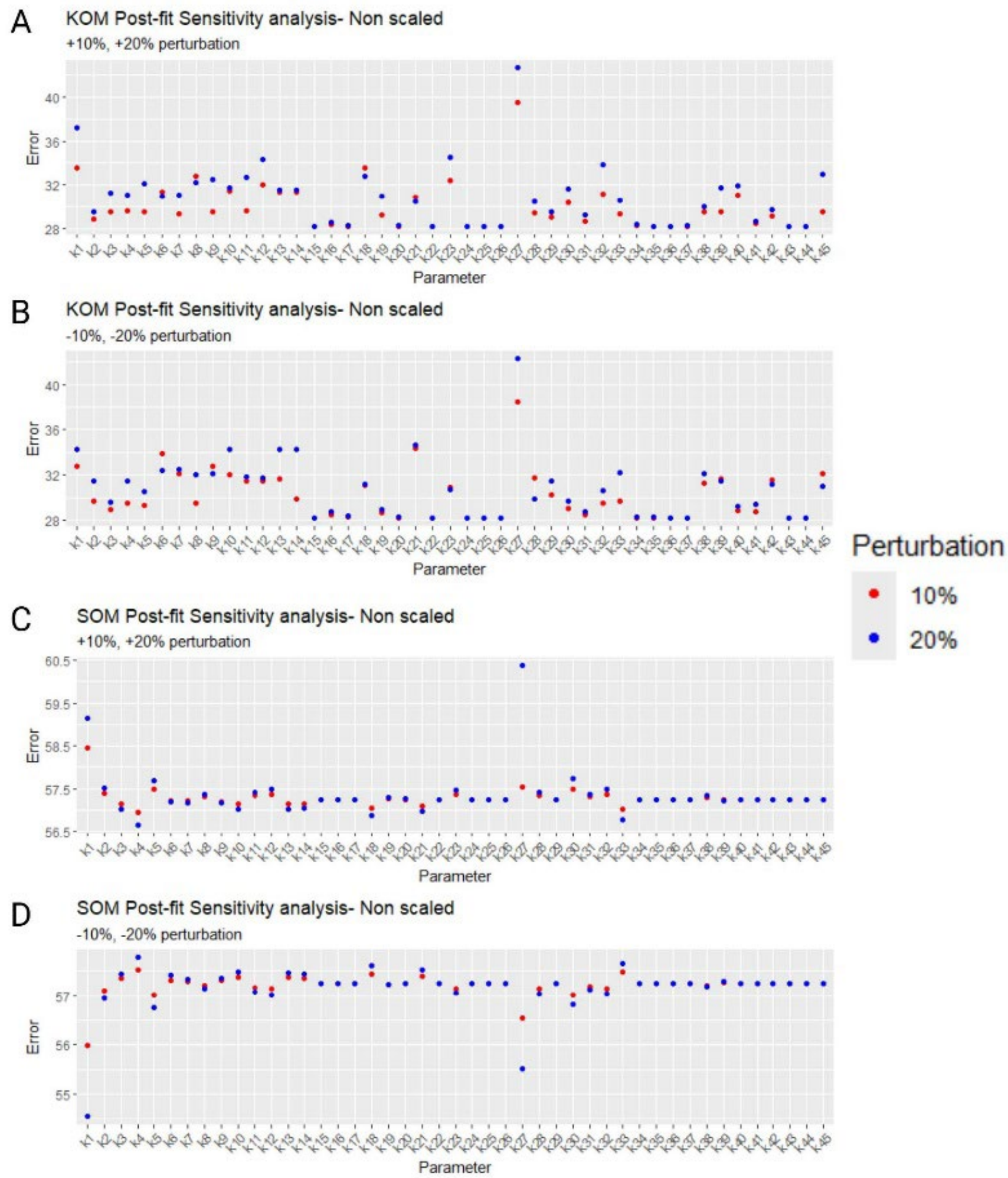

Supplementary Figure 5: Post-fit parameter sensitivity analysis. Each of the 45 parameters were perturbed by **(A)** +10% and 20% for the KOM, **(B)** -10% and 20% for the KOM, **(C)** +10% and 20% for the SOM, **(D)** -10% and 20% for the SOM and the resulting error values plotted. *Created in BioRender. Betenbaugh, M. (2026) https://BioRender.com/hkw7nyj*

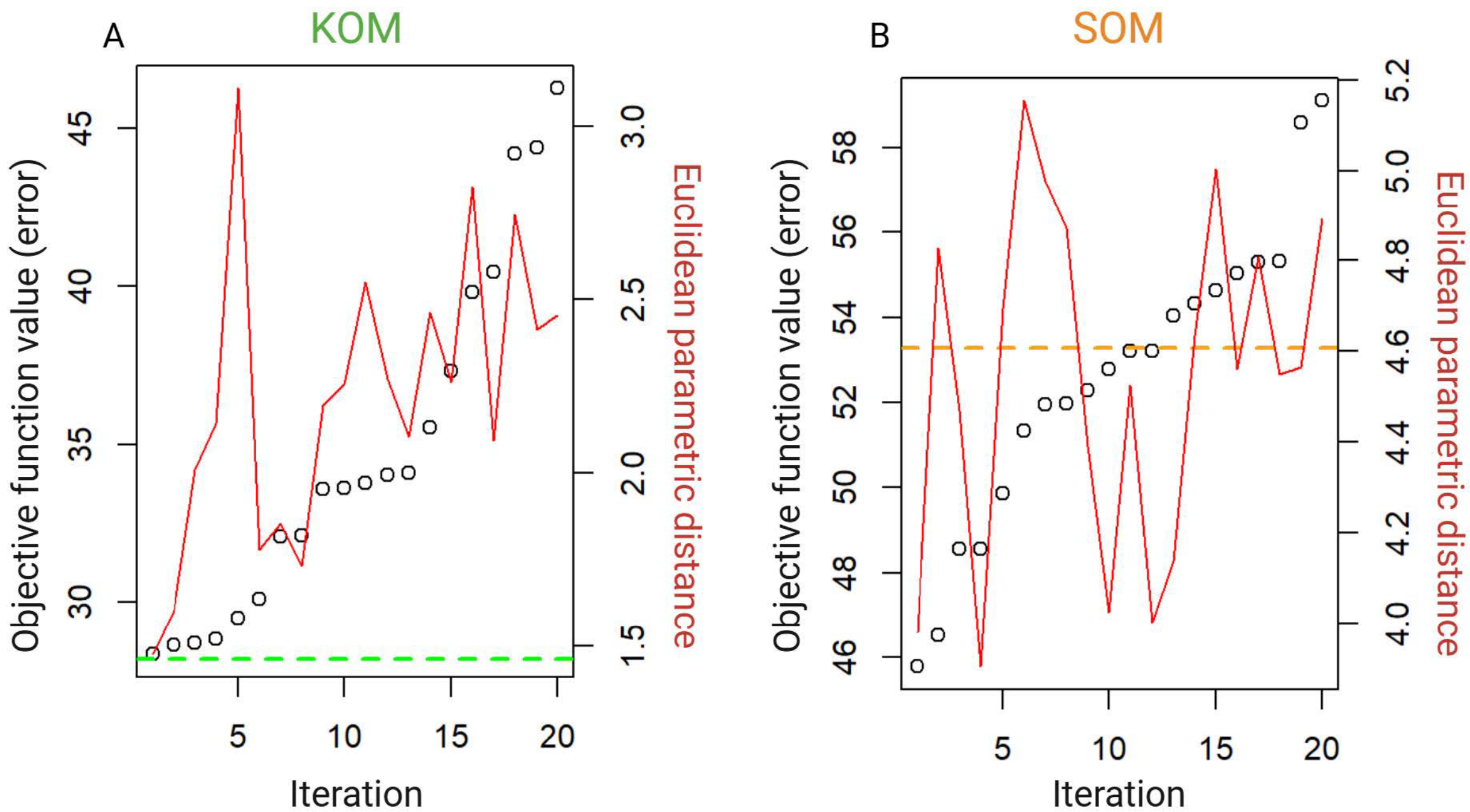

Supplementary Figure 6: MultiStart parameter sensitivity analysis. The initial parameter set was perturbed within ±20% of the original initial guess for **(A)** the kinetic-oriented model (KOM) and **(B)** the stoichiometric-oriented model (SOM). The distance of the final parameter set from the parameter set reported in the paper is shown (red line in both graphs), along with the final error values (hollow circles). Dashed lines in each graph indicate the final error value for the model reported in this paper. Graphs are arranged in ascending order of error.
Created in BioRender. Betenbaugh, M. (2026) https://BioRender.com/329o4bm

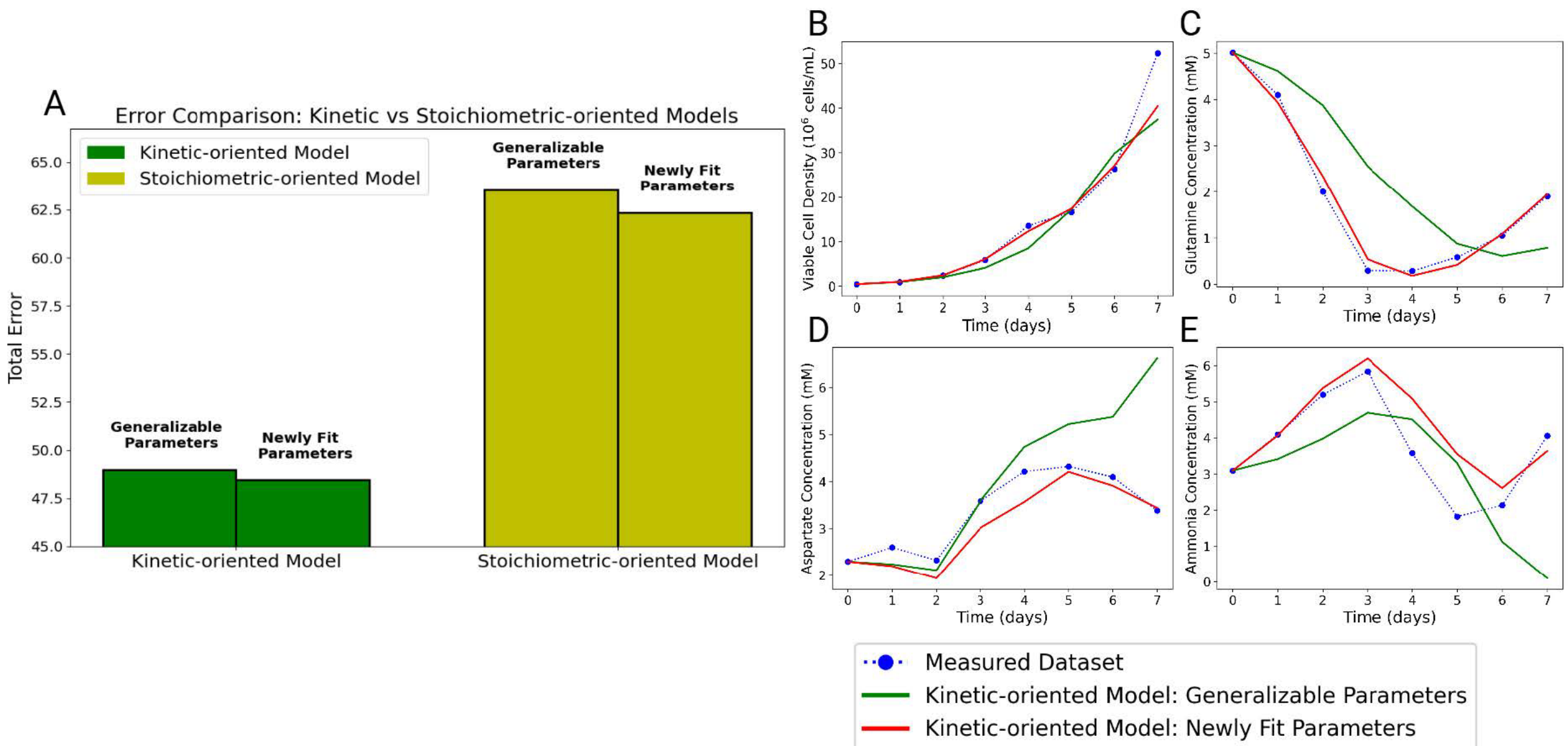

Supplementary Figure 7: Comparison of model fits and errors using parameters derived from the Modified Initial Conditions dataset. (A) Total simulation error for KOM and SOM with parameters from either the Control Case or the Modified Initial Conditions dataset, showing consistently lower error for KOM. **(B–E)** KOM predictions using control-case versus newly fit parameters for selected profiles: **(B)** Viable Cell Density (VCD), **(C)** Glutamine, **(D)** Aspartate, and **(E)** Ammonia. Newly fit parameters improved predictions for Glutamine, Aspartate, and Ammonia, while VCD remained similar between parameter sets.
Created in BioRender. Betenbaugh, M. (2026) https://BioRender.com/zqflpte

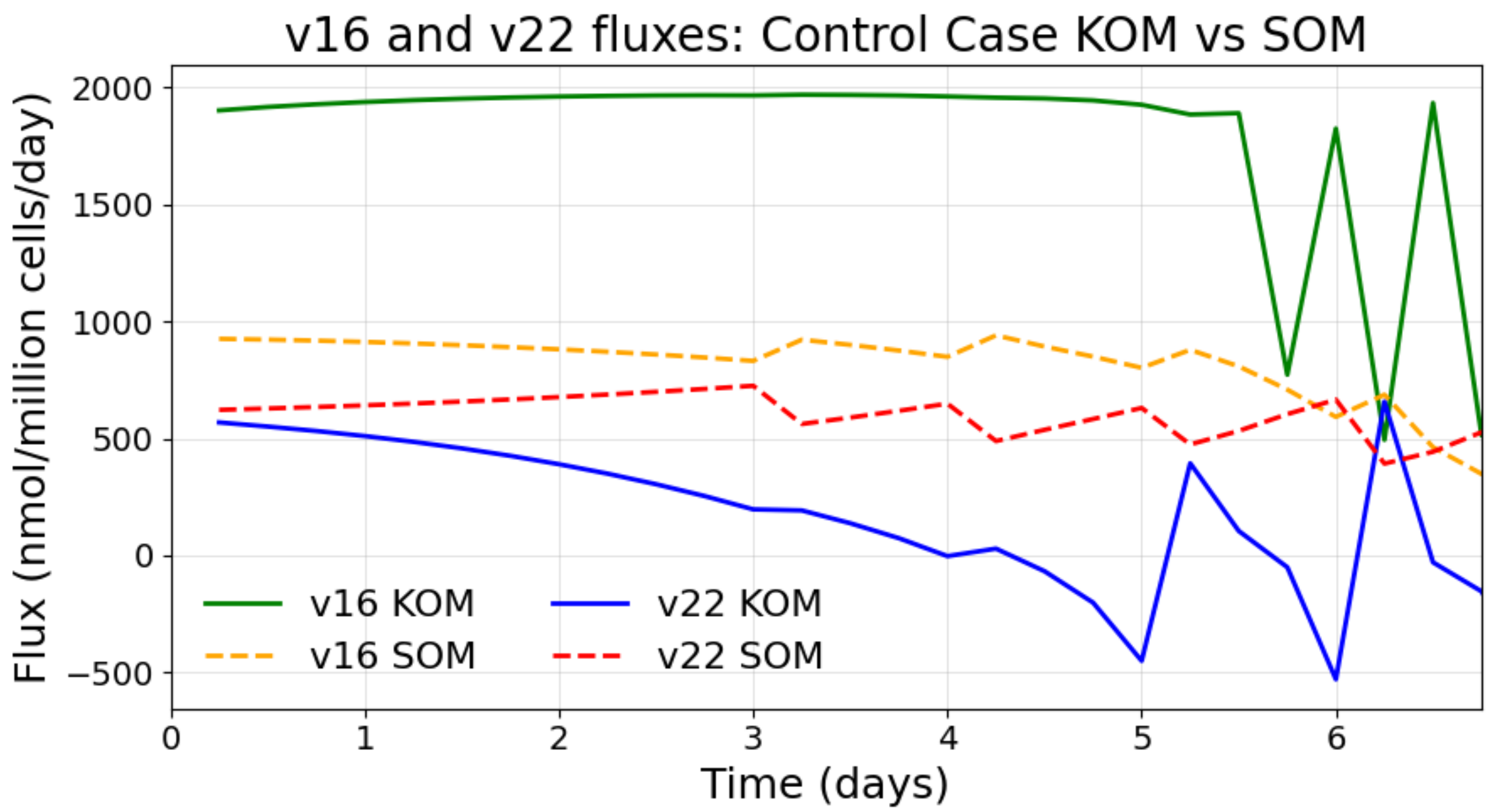

Supplementary Figure 8: Flux profiles for biomass generation (v16) and alanine generation/consumption (v22) in the Control Case. The KOM captures dynamic fluctuations in biomass (v16, green) and alanine (v22, blue) fluxes consistent with feeding-driven metabolite changes, while the SOM produces flat profiles for biomass (v16, yellow) and alanine (v22, red). Reduced biomass flux leads to underprediction of VCD, and persistently positive alanine flux results in continued accumulation, highlighting the inability of the SOM to capture dynamic culture behavior.

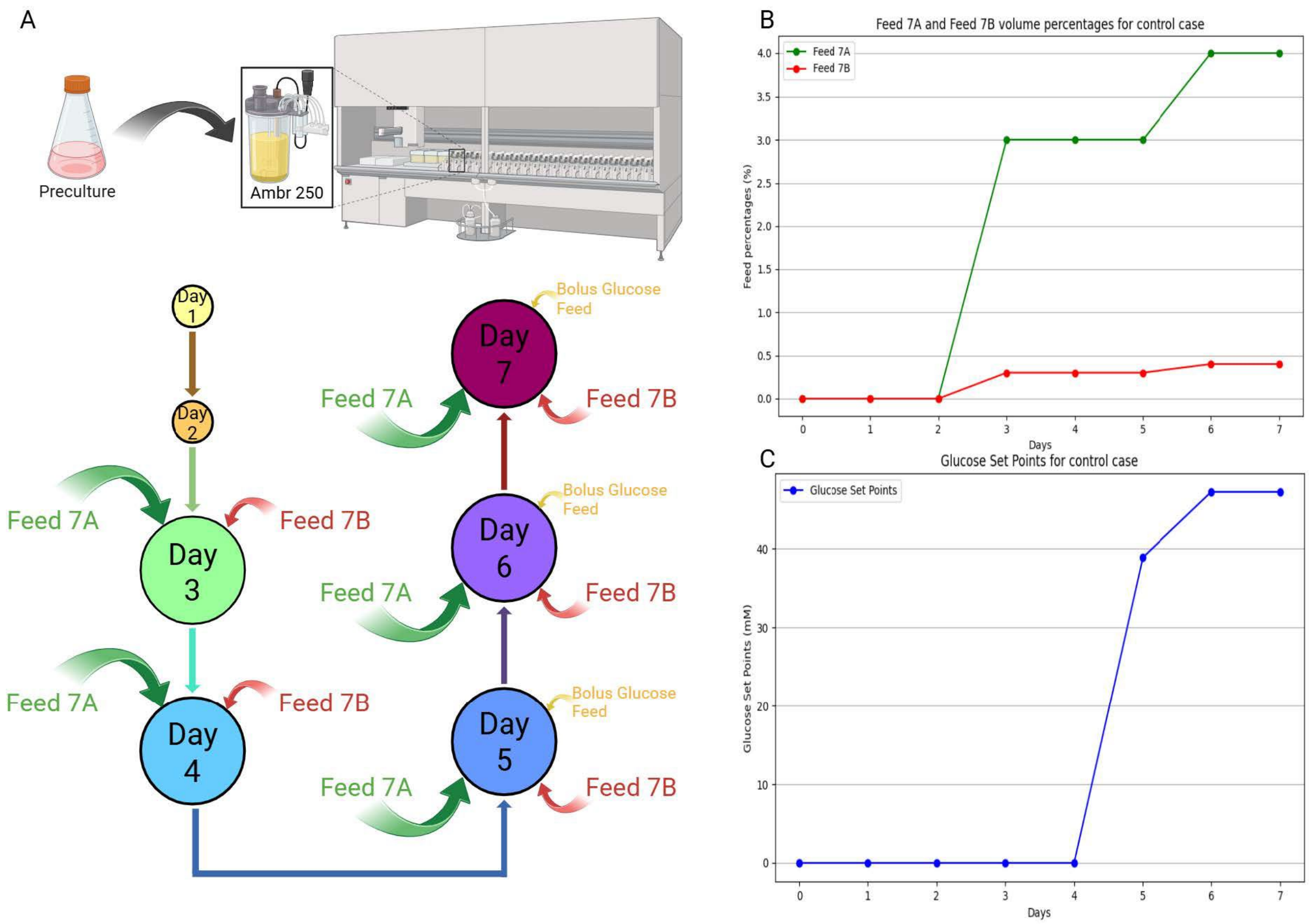

Supplementary Figure 9: Schematic of feeding strategy, feed concentrations, and glucose set points. (A) A schematic representation of the CHO cell culture from Day 0 to Day 7, highlighting the feeding strategy. Cells were precultured in shake flasks and subsequently transferred to an Ambr250 vessel. Feeding begins on Day 3 and includes Feed 7A, Feed 7B (Cell Boost 7a/7b- Cytiva), and bolus glucose additions. **(B)** Corresponding line graphs for Feed 7A and 7B concentrations. **(C)** Glucose set points.

Created in BioRender. Betenbaugh, M. (2026) https://BioRender.com/5mujt1l

Supplementary Table 1: Kinetic expressions for 14 reactions, including 12 intracellular reactions and 2 exchange fluxes, with their corresponding reaction indices. All expressions follow the Michaelis-Menten framework and are defined by extracellular concentrations of dynamically tracked variables together with kinetic parameters. The kinetic parameters include maximum reaction velocity (v_{max}), half-saturation constant (K_m), and inhibition constant (K_i).

Index number	Reaction	Kinetic expression
R1	G6P→2PYR+3ATP+ 2NADH	$v_1 = \frac{v_{max1} \cdot \left(\frac{GLC}{K_{m1}}\right)}{\left(1+\frac{LAC}{K_{i1}}\right) \cdot \left(1+\frac{GLC}{K_{m1}}\right)}$
R2	PYR + NADH → LAC	$v_2 = \frac{v_{max2f} \cdot \left[\left(\frac{GLC}{K_{m2glc}}\right)\right] - v_{max2r} \cdot \left(\frac{LAC}{K_{m2lac}}\right)}{1+\left(\frac{LAC}{K_{m2lac}}\right)+\left(\frac{GLC}{K_{m2glc}}\right)}$
R3	PYR+GLU→ALA+ AKG	$v_3 = \frac{v_{max3f} \cdot \left[\left(\frac{GLC}{K_{m3glc}}\right)\right] - v_{max3r} \cdot \left(\frac{ALA}{K_{m3ala}}\right)}{1+\left(\frac{GLC}{K_{m3glc}}\right)+\left(\frac{ALA}{K_{m3ala}}\right)}$
R8	GLN→GLU+NH3	$v_8 = \frac{v_{max8f} \cdot \left(\frac{GLN}{K_{m8gln}}\right) - v_{max8r} \cdot \left(\frac{GLU}{K_{m8glu}}\right)\left(\frac{NH3}{K_{m8nh3}}\right)}{1+\left(\frac{GLN}{K_{m8gln}}\right)+\left(\frac{GLU}{K_{m8glu}}\right)+\left(\frac{NH3}{K_{m8nh3}}\right)+\left(\frac{GLU}{K_{m8glu}}\right)\left(\frac{NH3}{K_{m8nh3}}\right)}$
R9	AKG+NH3+NADH→ GLU	$v_9 = \frac{v_{max9f} \cdot \left(\frac{NH3}{K_{m9nh3}}\right) - v_{max9r} \cdot \left[\left(\frac{GLU}{K_{m9glu}}\right)+\left(\frac{GLN}{K_{m9gln}}\right)\right]}{1+\frac{NH3}{K_{m9nh3}}+\frac{GLU}{K_{m9glu}}+\frac{GLN}{K_{m9gln}}+\left(\frac{GLU}{K_{m9glu}}\right)\left(\frac{GLN}{K_{m9gln}}\right)}$
R10	ASN→ASP+NH3	$v_{10} = \frac{vmax_{10f} \cdot \left(\frac{ASN}{K_{m10asn}}\right)}{1+\left(\frac{ASN}{K_{m10asn}}\right)}$
R11	ASP+AKG→OXA+ GLU	$v_{11} = \frac{v_{max11f} \cdot \left[\left(\frac{ASP}{K_{m11asp}}\right)+\left(\frac{ASN}{K_{m11asn}}\right)\right] - v_{max11r}\left(\frac{GLU}{K_{m11glu}}\right)}{1+\left(\frac{ASP}{K_{m11asp}}\right)+\left(\frac{ASN}{K_{m11asn}}\right)+\left(\frac{ASP}{K_{m11asp}}\right)\left(\frac{ASN}{K_{m11asn}}\right)}$
R12	SER+CO2+NH3+NADH→2 GLY	$v_{12} = \frac{v_{max12f} \cdot \left(\frac{SER}{K_{m12ser}}\right)\left(\frac{NH3}{K_{m12nh3}}\right) - v_{max12r} \cdot \left(\frac{GLY}{K_{m12gly}}\right)^2}{1+\left(\frac{SER}{K_{m12ser}}\right)+\left(\frac{NH3}{K_{m12nh3}}\right)+\left(\frac{GLY}{K_{m12gly}}\right)+\left(\frac{GLY}{K_{m12gly}}\right)^2}$
R13	Cystine+NADH→ 2Cysteine	$v_{13} = \frac{v_{max13} \cdot \left(\frac{C-C}{K_{m13}}\right)}{\left(1+\left(\frac{C-C}{K_{m13}}\right)\right)}$
R16	Biomass production	$v_{16} = \frac{v_{max16} \cdot \left(\frac{GLN}{K_{m16a}}\right) \cdot \left(\frac{ASN}{K_{m16b}}\right) \cdot \left(\frac{ALA}{K_{m16c}}\right)}{1+\left(\frac{GLN}{K_{m16a}}\right)+\left(\frac{ASN}{K_{m16b}}\right)+\left(\frac{ALA}{K_{m16c}}\right)+\left(\frac{GLN}{K_{m16a}}\right) \cdot \left(\frac{ASN}{K_{m16b}}\right)+\left(\frac{GLN}{K_{m16a}}\right) \cdot \left(\frac{ALA}{K_{m16c}}\right)+\left(\frac{ASN}{K_{m16b}}\right) \cdot \left(\frac{ALA}{K_{m16c}}\right)+\left(\frac{GLN}{K_{m16a}}\right) \cdot \left(\frac{ASN}{K_{m16b}}\right) \cdot \left(\frac{ALA}{K_{m16c}}\right)}$
R17	Antibody synthesis	$v_{17} = \frac{v_{max17}}{1+\left(\frac{LAC}{K_{i17}}\right)}$

R18	Biomass exchange reaction	$v_{18} = v_{16}$
R19	Antibody exchange reaction	$v_{19} = v_{17}$
R35	G6P + ATP + 2GLU→ 2NADH + 2SER + 2AKG	$v_{35} = \frac{v_{max35} \cdot \left(\frac{GLC}{K_{m35a}}\right) \cdot \left(\frac{GLU}{K_{m35b}}\right)^2}{\left(1 + \left(\frac{GLC}{K_{m35}}\right) + \left(\frac{GLU}{K_{m35b}}\right) + \left(\frac{GLU}{K_{m35b}}\right)^2\right)}$

Supplementary Table 2: Reaction expressions for all 35 reactions in the model. Extracellular metabolites are denoted with the subscript "E". NADH is separated into cytosolic and mitochondrial pools, represented by the subscripts "cyto" and "mito", respectively.

1. G6P → 2 PYR + 3 ATP + 2 $NADH_{cyto}$
2. PYR + $NADH_{cyto}$ ↔ LAC
3. PYR + GLU ↔ ALA + AKG
4. PYR + OXA → AKG + 2 CO_2 + 2 $NADH_{mito}$
5. AKG → MAL + CO2 + $NADH_{mito}$ + FADH2 + ATP
6. MAL → OXA + $NADH_{mito}$
7. MAL → PYR + CO_2
8. GLN ↔ GLU + NH_3
9. AKG + NH_3 + $NADH_{mito}$ ↔ GLU
10. ASN → ASP + NH_3
11. ASP + AKG ↔ OXA + GLU
12. SER + CO_2 + NH_3 + $NADH_{cyto}$ ↔ 2 GLY
13. Cystine + $NADH_{cyto}$ → 2 Cysteine
14. $NADH_{mito}$ + 0.5 O_2 → 2.5 ATP
15. $FADH_2$ + 0.5 O_2 → 1.5 ATP
16. 0.0838 ALA + 0.041 ASN + 0.0804 ASP + 8.6825 ATP + 0.0261 Cysteine + 0.452 G6P + 0.0873 GLN + 0.056 GLY + 0.427 OXA + 0.096 SER → BIOM + 0.004 $FADH_2$ + 0.0082 GLU + 0.4445 MAL + 0.6391 $NADH_{mito}$ + 0.2085 PYR
17. 0.0614 ALA + 0.0344 ASN + 0.0389 ASP + 9.2 ATP + 0.024 Cysteine + 0.0479 GLU + 0.0449 GLN + 0.0719 GLY + 0.1 SER → ANTI
18. BIOM → $BIOM_E$
19. ANTI → $ANTI_E$
20. GLC_E + ATP → G6P
21. LAC ↔ LAC_E
22. ALA ↔ ALA_E
23. ASN_E → ASN
24. ASP ↔ ASP_E
25. $Cystine_E$ + GLU → Cystine + GLU_E
26. GLN_E ↔ GLN
27. GLY ↔ GLY_E
28. SER_E ↔ SER
29. NH3 ↔ $NH3_E$
30. $O2_E$ → O2
31. CO2 → $CO2_E$
32. 2 Cysteine + O2 → Cystine
33. GLU ↔ GLU_E
34. $NADH_{cyto}$ → 0.5 $NADH_{mito}$ + 0.5 $FADH_2$
35. G6P + ATP + 2 GLU → 2 $NADH_{cyto}$ + 2 SER + 2 AKG

Supplementary Table 3: Mass balances for all dynamically tracked species were modeled using forward Euler ODEs. Overall cell density was calculated from biomass, while a separate ODE described dead cell density.

**Numerical Integration and Biomass Conversion Formulas**
Forward Euler equations for metabolite species: $$C_{k,i} = C_{k,i-1} + \frac{v_{k,i-1}(t_i - t_{i-1})VCD_{i-1}}{1000} \quad (1)$$ C = Concentration (mM)<br>k = metabolite species or Biomass or Antibody<br>VCD = viable cell density (million cells/mL)<br>v = exchange flux (nmol/million cells/day)<br>t_i = i^{th} timepoint (days)
Overall cell density calculated as: $$Overall\ cell\ density = \frac{Biomass}{2.31} \quad (2)$$ Since it was experimentally determined that 1 million cells is equivalent to 2.31mM of biomass Nolan et al.
Dead cell density calculated as: $$Dead\ cell\ density_i = Dead\ cell\ density_{i-1} + k_d VCD_{i-1} \quad (3)$$ k_d = Death rate constant

<u>Supplementary Table 4:</u> Rationale behind the formulation and modification of kinetic expressions mentioned in Supplementary Table 1. The expressions were either modified from previous work (Nolan et al.[13]) or added based on experimental data.

Reaction	Modification of kinetic expression
1	Temperature dependent and inhibition exponential constant (which is also temperature dependent) were removed from the expression.
2	Given the abrupt discontinuity introduced due to the redox variable, it was removed. What was originally two distinct expressions dependent on the redox variable, was made one expression. The forward rate is governed by the corresponding extracellular metabolites producing PYR, i.e., GLC and the reverse rate, dependent on extracellular LAC.
3	Similar to the reaction above, the redox variable was removed.
8	Temperature dependent constant was removed.
9	While the original model built in the dependence of AKG via reaction 3 and the dependence of intracellular GLU on reaction 11, it disrupts the typical Michaelis-Menten format of rate expressions. Thus, the forward reaction was made dependent on NH3 (the only metabolite with an extracellular component driving the forward reaction) and the reverse reaction made dependent on extracellular GLU and GLN which drive the production of intracellular GLU.
10	Temperature dependent constant was removed. The stoichiometry of the reaction was revised to make it irreversible, based on experimentally collected ^{13}C data, and correspondingly, the kinetic expression was modified to remove the reverse reaction component.
11	Reaction was modified to make it more explicit in terms of the concentrations of the substrates and to make it adhere more closely to the Michaelis Menten format. Given that extracellular aspartate and asparagine both contribute to the pool of intracellular aspartate, the forward expression is dependent on both of these amino acids.
12	The reaction was made explicitly dependent on NH3 in addition to SER. The contributions of CO2 and NADH are considered insignificant compared to the other metabolites.
13	Redox variable was removed
16	Temperature constants and temperature dependency were removed. Biomass generation depends crucially on GLN and ASN as in the original model. Beyond these, multiple formulations of the biomass synthesis expression were evaluated for other components based on published literature and empirical model performance. An appropriate formulation was chosen by considering the inclusion of relevant metabolites and amino acids that are most closely related to biomass generation.
17	Exponential inhibition constant was removed.
35	Reaction was added to the stoichiometric network based on the ^{13}C-labeled metabolite tracking data which shows the formation of labeled serine from labeled glucose, glutamate and glutamine, indicating the presence of the serine synthesis pathway (SSP) originating from glycolysis and ending in serine (supported by literature).

Supplementary Table 5: Flux optimization formulations and numerical solvers for the SOM and KOM

	SOM	KOM
**Condition enforced**	$S.v = 0$	Selected fluxes fixed by kinetic expressions
**Constraint minimized**	$\Sigma(v_{k,st} - v_{k,kin})^2$	$S.v = 0$
**Bounds**	$Irreversible: [lb, ub] = [0, +\infty]$<br>$Reversible: [lb, ub] = [-\infty, +\infty]$	
**Numerical tool**	Nonlinear constrained optimizer (fmincon)	Linear least-squares solver (lsqlin)

Supplementary Table 6: Optimized parameter values for the kinetic-oriented (KOM) and stoichiometric-oriented (SOM) models derived from the control case

Parameter index	Parameter	KOM value	SOM value
k_1	v_{max1}	2039.3	673.09
k_2	K_{i1}	58.21	19.97
k_3	K_{m1}	2.63	11.61
k_4	v_{max2f}	7034.18	3697.81
k_5	K_{m2glc}	3.55	4.23
k_6	v_{max3f}	858.74	910.66
k_7	v_{max3r}	395.92	62.17
k_8	K_{m3ala}	0.23	9.78
k_9	K_{m3glc}	1.55	0.7
k_{10}	v_{max8f}	4027.44	7175.67
k_{11}	v_{max8r}	346.93	104.15
k_{12}	K_{m8gln}	1.93	0.67
k_{13}	K_{m8glu}	0.96	0.9
k_{14}	K_{m8nh3}	1.13	0.87
k_{15}	v_{max9f}	0.49	2.23
k_{16}	v_{max9r}	6.74	7.56
k_{17}	K_{m9nh3}	0.35	0.03
k_{18}	v_{max10f}	68.59	76.38
k_{19}	K_{m10asn}	0.44	0.996
k_{20}	v_{max11f}	0.27	0.15
k_{21}	v_{max12f}	0.98	0.99
k_{22}	v_{max12r}	22.89	7.12
k_{23}	K_{m12ser}	0.1	0.1
k_{24}	K_{m12gly}	2.19	1.34
k_{25}	v_{max13}	21.39	8.63
k_{26}	K_{m13}	1.61	0.92
k_{27}	v_{max16}	2022.25	843.72
k_{28}	K_{m16a}	0.03	0.13
k_{29}	K_{m16b}	0.03	0.05

k_{30}	v_{max17}	128.63	116.74
k_{31}	K_{i17}	19.12	10.51
k_{32}	v_{max2r}	1302.23	931.88
k_{33}	K_{m2lac}	0.32	0.93
k_{34}	K_{m9glu}	0.3	0.1
k_{35}	K_{m9gln}	2.26	2.56
k_{36}	K_{m11asp}	2.37	1.88
k_{37}	K_{m11asn}	1.46	0.78
k_{38}	K_{m12nh3}	0.01	0.01
k_{39}	K_{m16c}	0.02	0.01
k_{40}	v_{max35}	11.41	9.58
k_{41}	K_{m35a}	2.45	0.11
k_{42}	K_{m35b}	3.79	0.28
k_{43}	v_{max11r}	0.22	0.08
k_{44}	K_{m11glu}	1.6	0.98
k_{45}	k_d	0.0049	0.0003

Units for maximum reaction velocities (v_{max}) are nmol/10^6 cells/day, half-saturation constant (K_m) are mM and cell death rate is dimensionless

Supplementary Table 7: Measured concentrations of metabolites, amino acids, antibody, and cell densities for the control case from days 0-7.

Measured Concentrations/Quantities of Metabolites								
**Metabolite/species**	**Days**							
	**0**	**1**	**2**	**3**	**4**	**5**	**6**	**7**
Antibody (g/L)	0.01	0.01	0.01	0.1±0.0	0.16± 0.01	0.28± 0.00	0.46± 0.02	0.71± 0.02
Glucose (mM)	30.9±0.2	28.61± 4.23	24.83± 1.08	31.97± 3.01	37.73± 0.43	35.01± 0.22	39.25±1.7	55.69± 1.41
Lactate (mM)	1.33	2.55± 0.13	4.4± 0.3	12.88± 0.28	10.44± 0.36	2.74± 0.36	2.33± 0.35	4.22± 0.15
Alanine (mM)	0.35± 0.14	0.66± 0.14	1.47± 0.17	2.77± 0.27	3.18± 0.43	1.68± 0.28	0.63± 0.08	0.62± 0.04
Asparagine (mM)	5.23± 0.22	4.84± 0.87	4.68± 0.45	5.82± 0.55	4.97± 0.82	5.28± 0.52	4.21± 0.55	1.66± 0.17
Aspartate (mM)	1.21± 0.18	1.31± 0.07	1.91± 0.12	3.55± 0.38	3.51±0.7	4.64± 0.28	6.21± 1.05	3.99± 0.45
Cystine (mM)	0.332± 0.092	0.44± 0.04	0.26± 0.02	0.87± 0.09	1.1±0.09	1.27± 0.03	1.04± 0.09	0.37± 0.01
Glutamine (mM)	4.36± 0.01	2.05± 0.31	0.58± 0.06	0.14±0	0.29± 0.04	0.52±0	0.88± 0.04	1.48±0
Glycine (mM)	1.12± 0.03	1.21± 0.51	1.86± 0.18	3.47± 0.65	2.38±0.1	3.32± 0.45	4.14± 1.34	2.62± 1.07
Serine (mM)	7.76± 0.72	7.45± 0.9	6.67± 0.39	7.72± 0.21	6.68± 0.57	7.38± 0.21	7.6± 0.7	6.86± 0.52
Ammonia (mM)	3.16± 0.02	2.8±0.3	3.24± 0.05	4.65± 0.08	2.69± 0.06	1.58± 0.04	1.73± 0.32	3.6± 0.5
Glutamate (mM)	2.67± 0.01	1.75± 0.24	1.34± 0.05	4.7±0.1	6.57± 0.64	7.29± 0.16	6.63± 0.26	5.83± 0.06
Viable cell density-VCD (million cells/mL)	0.495± 0.005	0.77± 0.08	2.59± 0.14	5.31± 0.05	10.66± 0.32	18.3± 0.25	32.3± 0.1	44.2± 4.6

Values are presented as mean ± SEM. Number of biological replicates = 3 for most datapoints. For time points with a single replicate, only the individual value is shown.

Supplementary Table 8: Measured concentrations of metabolites, amino acids, antibody, and cell densities for the "modified initial conditions" case from days 0-7.

Measured Concentrations/Quantities of Metabolites								
**Metabolite/species**	**Days**							
	**0**	**1**	**2**	**3**	**4**	**5**	**6**	**7**
Antibody (g/L)	0.01	0.01	0.01	0.09576	0.14	0.27± 0.00	0.43± 0.01	0.72± 0.01
Glucose (mM)	29.7±0.9	29.07± 0.2	23.35± 0.3	17.2± 0.12	30.09± 0.49	28.28± 0.08	24.8±0.9	19.74± 0.64
Lactate (mM)	1.26± 0.04	3.44± 0.13	N/A	14.88± 0.23	N/A	10.4± 0.2	N/A	2.22± 0.11
Alanine (mM)	0.28± 0.02	0.7± 0.01	1.28± 0.06	2.57± 0.08	2.97± 0.09	1.4±0.06	0.42± 0.01	0.39± 0.01
Asparagine (mM)	4.47± 0.11	4.31± 0.1	4.04± 0.03	4.7±0.1	5.07± 0.06	3.99± 0.02	2.31± 0.04	0.84± 0.06
Aspartate (mM)	2.29± 0.05	2.6± 0.26	2.32± 0.06	3.58± 0.06	4.21± 0.25	4.32± 0.58	4.09± 0.96	3.38± 0.86
Cystine (mM)	1.49± 0.54	1.54± 0.57	1.67± 0.71	3.03± 1.11	3.44±1.2	4.1±1.4	4.21± 1.51	3.32± 1.49
Glutamine (mM)	5.01± 0.13	4.1± 0.01	2±0.0	0.29± 0.01	0.28±0	0.58± 0.01	1.05±0	1.9±0.01
Glycine (mM)	0.609± 0.424	0.64± 0.51	0.81± 0.7	0.89± 0.66	1.38± 0.89	0.7±0.43	0.49± 0.29	0.25± 0.08
Serine (mM)	2.98± 2.69	3.05± 2.58	2.56± 2.3	2.9± 2.0	3.14± 2.02	2.57± 1.33	2.16± 1.19	1.57± 1.18
Ammonia (mM)	3.09± 0.02	4.09±0.01	5.19±0.06	5.84±0.03	3.57±0.02	1.82±0.01	2.13±0.08	4.05±0.11
Glutamate (mM)	2.45± 0.075	2.57±0.01	2.47±0.05	2.66±0.04	4.3±0.07	5.47±0.04	5.15±0.14	3.57±0.08
Viable cell density- VCD (million cells/mL)	0.452± 0.015	0.86±0.02	2.38±0.03	5.88±0.28	13.53± 0.15	16.58±1.27	26.34±1.16	52.27±0.57

Values are presented as mean ± SEM. Number of biological replicates = 3 for most datapoints. For time points with a single replicate, only the individual value is shown. N/A indicates that no measurement was made at that time point.

Supplementary Table 9: Measured concentrations of metabolites, amino acids, antibody, and cell densities for the "lactate supplemented" case from days 0-7.

Measured Concentrations/Quantities of Metabolites								
**Metabolite/species**	**Days**							
	0	1	2	3	4	5	6	7
Antibody (g/L)	0.01	0.03	0.05	0.07	0.085± 0.005	0.135± 0.005	0.2±0.01	0.29
Glucose (mM)	29.1± 0.4	26.86± 2.81	13.81	25.14± 0.08	35.39± 0.89	39.56± 1.06	42.44± 1.33	44.86± 1.69
Lactate (mM)	1.22	25.7± 1.17	N/A	34.75± 3.44	N/A	25.98± 0.22	N/A	4.94± 0.94
Alanine (mM)	N/A	N/A	N/A	N/A	N/A	N/A	N/A	N/A
Asparagine (mM)	N/A	N/A	N/A	N/A	N/A	N/A	N/A	N/A
Aspartate (mM)	N/A	N/A	N/A	N/A	N/A	N/A	N/A	N/A
Cystine (mM)	N/A	N/A	N/A	N/A	N/A	N/A	N/A	N/A
Glutamine (mM)	4.995± 0.025	3.91± 0.39	2.51± 0.09	1.34± 0.2	0.41± 0.02	0.45± 0.01	0.9±0.01	1.67± 0.06
Glycine (mM)	N/A	N/A	N/A	N/A	N/A	N/A	N/A	N/A
Serine (mM)	N/A	N/A	N/A	N/A	N/A	N/A	N/A	N/A
Ammonia (mM)	3.09	3.91± 0.06	4.42± 0.14	4.59± 0.38	4.06± 0.16	3±0.1	2.91±0	4.94± 0.1
Glutamate (mM)	2.4	2.36± 0.21	2.33± 0.12	2.29± 0.27	4.31± 0.04	6.24± 0.15	7.37± 0.11	8.42± 0.56
Viable cell density-VCD (million cells/mL)	0.395 ± 0.054	0.79± 0.01	1.77± 0.04	3.81± 0.01	6.32± 0.22	10.41± 0.11	10.58±0.33	22.3±0.4

Values are presented as mean ± SEM. Number of biological replicates = 3 for most datapoints. For time points with a single replicate, only the individual value is shown. N/A indicates that no measurement was made at that time point.

Supplementary Table 10:

Timepoint (days)	Fluxes for biomass generation and alanine production/consumption			
	Kinetic-oriented model		Stoichiometric-oriented model	
	v16 (Biomass)	v22 (Alanine)	v16 (Biomass)	v22 (Alanine)
0.25	1902.75881	568.766402	926.1325	622.4039
0.5	1917.54014	551.1775735	922.8625	628.4039
0.75	1929.10597	532.1731374	918.4553	634.7456
1	1938.68794	510.511173	912.9556	641.7611
1.25	1946.57271	485.8383579	906.461	649.4838
1.5	1952.98885	457.7788081	899.0158	657.9429
1.75	1958.11387	425.9740235	890.6229	667.1657
2	1962.07746	390.0703879	881.2488	677.1773
2.25	1964.95975	349.7056774	870.8234	687.9992
2.5	1966.78315	304.4755899	859.2348	699.6447
2.75	1967.49319	253.8617468	846.3195	712.1111
3	1966.91826	197.088443	831.8425	725.3644
3.25	1969.9147	192.5624972	922.343	562.1384
3.5	1968.90104	137.6963393	900.3383	589.5616
3.75	1966.66259	73.80820885	876.1293	618.5277
4	1962.44631	-2.87612554	849.1936	648.8931
4.25	1957.40174	29.12342583	941.0681	489.1707
4.5	1953.90091	-67.4800315	893.2149	537.202
4.75	1945.5732	-201.808235	850.1037	583.3925
5	1926.94057	-450.552445	802.2661	630.6819
5.25	1885.26551	393.9662387	879.255	474.5714
5.5	1890.93178	104.5555484	808.4232	532.5969
5.75	772.913055	-50.5703094	708.887	604.2199
6	1825.10499	-529.401199	592.3594	666.2646
6.25	494.299326	657.5740704	687.3929	392.5595
6.5	1934.52876	-29.2845556	463.0169	443.3808
6.75	513.463542	-153.695668	348.5713	526.9391
7	1783.68125	-528.63431	285.5604	606.7188

Supplementary Table 11: Measured initial concentrations of metabolites, amino acids, antibody, and cell densities for each of the three experiments: control case, modified initial conditions case, and lactate-supplemented case.

**Measured Initial Concentrations/Quantities of Metabolites**			
**Metabolite/species**	**Control Case**	**Modified Initial Conditions: Alternate Dataset 1**	**Lactate-Supplemented: Alternate Dataset 2**
Antibody (g/L)	0.01	0.01	0.01
Glucose (mM)	30.9 ± 0.2	29.7 ± 0.9	29.1 ± 0.4
Lactate (mM)	1.33	1.26 ± 0.04	1.22
Alanine (mM)	0.35 ± 0.14	0.28 ± 0.02	N/A
Asparagine (mM)	5.23 ± 0.22	4.47 ± 0.11	N/A
Aspartate (mM)	1.21 ± 0.18	2.29 ± 0.05	N/A
Cystine (mM)	0.332 ± 0.092	1.49 ± 0.54	N/A
Glutamine (mM)	4.36 ± 0.01	5.01 ± 0.13	4.995 ± 0.025
Glycine (mM)	1.12 ± 0.03	0.609 ± 0.424	N/A
Serine (mM)	7.76 ± 0.72	2.98 ± 2.69	N/A
Ammonia (mM)	3.16 ± 0.02	3.09 ± 0.02	3.09
Glutamate (mM)	2.67 ± 0.01	2.45 ± 0.075	2.4
Viable cell density- VCD (million cells/mL)	0.495 ± 0.005	0.452 ± 0.015	0.395 ± 0.054

Values are presented as mean ± SEM when $n \geq 2$; for timepoints with a single replicate, only the individual value is shown. In the lactate-supplemented condition, some amino acids were not measured and are indicated as N/A.

Supplementary Table 12: Concentrations of select model-relevant metabolites in each of the three feed streams.

Concentrations/Quantities (mM) Of Metabolites in Feeds			
**Metabolites**	**Feed 7A**	**Feed 7B**	**Bolus Glucose**
Glucose	360.8		2500
Alanine	21.8		
Asparagine	35		
Aspartate	62.3		
Cysteine/Cystine	0	85.00	
Glutamine	0		
Glycine	0		
Serine	110.5		
Ammonia	0		
Glutamate	100.6		

<u>Supplementary Table 13</u>: PID settings for DO control in the ambr250 HT bioreactor system. Level 0 represents the initial conditions for the control manipulated variables. Level 1 increases the total gas flow rate when the DO falls below the DO set-point. No overlay gas was used. Air, O_2, and CO_2 are all provided through the sparge gas stream.

Level	Variable	Minimum	Maximum	K_p	t_I (s)	t_D (s)
0	Stir speed (rpm)<br>Gas flow rate (mL/min)<br>O_2 mix (%) *<br>CO_2 flow rate (mL/min)	300<br>2<br>0<br>0.1	300<br>2<br>0<br>0.1	NA	NA	NA
1	Total gas flow rate (mL/min)	2	20	0.10	0	100
2	O_2 mix (%)	0	50	0.15	0	100
3	Stir speed (rpm)	300	400	2.0	0	100
4	O_2 mix (%)	50	100	0.15	0	250
5	Stir speed (rpm)	400	800	1.0	0	100
6	O_2 flow (mL/min)	0	20	0.50	0	100

*O_2 mix is the percentage of oxygen mixed with the sparge gas, which contains air and a variable amount of carbon dioxide that is used for pH control. NA – Not Applicable
